# Effects of short-term, sublethal fipronil and its metabolite on dragonfly feeding activity

**DOI:** 10.1371/journal.pone.0200299

**Published:** 2018-07-11

**Authors:** Hiroshi Jinguji, Kazuhisa Ohtsu, Tetsuyuki Ueda, Koichi Goka

**Affiliations:** 1 School of Food, Agricultural and Environmental Sciences, Miyagi University, Sendai, Miyagi, Japan; 2 Division of Biodiversity, Chemical Substances Effect Assessment Unit, National Agriculture and Food Research Organization, Tsukuba, Ibaraki, Japan; 3 Environmental Sciences, Ishikawa Prefectural College, Ishikawa, Japan; 4 National Institute for Environmental Sciences, Tsukuba, Ibaraki, Japan; University of California San Diego, UNITED STATES

## Abstract

Dragonflies, *Sympetrum* spp., are indispensable to agriculture and are a central element of culture in Japan. However, *S*. *frequens* populations in rice paddy fields have declined in recent decades. Dragonfly larvae are predatory aquatic insects that feed on other organisms found in habitats with slow-moving or standing water. The increasing use of fipronil and neonicotinoid insecticides in agriculture is also increasing exposure to *Sympetrum* spp. in larval stages through paddy soil and water. The role of fipronil insecticides in the decline of dragonflies is of concern, and we here examine the sublethal effects of this insecticide on the feeding behaviors of two *Sympetrum* spp. Based on the quantity of prey items consumed and the time to capture prey items, feeding inhibition was determined to be a potential mechanism of the decline of *Sympetrum* spp. following 48-h exposure to fipronil and fipronil sulfone. Prey consumption by *S*. *infuscatum* was significantly reduced for fipronil sulfone at all concentrations (0.01–1000 μg/L). *S*. *frequens* exposed to 1, 10, 100 and 1000 μg/L fipronil sulfone had significantly longer prey capture times. Fipronil sulfone was 2.8, 9.7 and 10.5 times more toxic to *S*. *infuscatum* than fipronil in terms of acute toxicity, feeding inhibition and delayed toxicity, respectively. In addition, fipronil sulfone was 6.6, 2.9 and 9.1 times more toxic, respectively, to *S*. *frequens* than fipronil. Our findings suggest that sublethal effects on feeding inhibition lead to severe mortality at realistic paddy soil and water concentrations. Our results provide the first demonstration that short-term exposure to fipronil and fipronil sulfone can consequently cause significant harm to dragonfly larvae survival due to feeding inhibition. These findings have implications for current pesticide risk assessment and dragonfly protection.

## Introduction

The use of insecticides in agriculture is increasing worldwide [1] and is increasing the exposure of non-target aquatic animals through processes like runoff in paddy fields. Dragonflies (*Sympetrum* spp.) are indispensable to agriculture and are a central element of culture in Japan [2]. *Sympetrum frequens* is important for several reasons. This species is one of the most effective predators of rice insect pests, such as the beetles *Lissorhoptrus oryzophilus* and *Oulema oryzae*, partly because of the high density of this dragonfly species in rice fields during the rice-growing season [3]. *S*. *frequens* is also a major predator of the mosquito *Anopheles sinensis*, a known malaria vector [3], and increased predation pressure by *S*. *frequens* may reduce the potential for malaria transmission in areas where the disease is endemic (e.g., Southeast Asia). Further, the many brands of “red dragonfly rice” on the market attest to the fact that *S*. *frequens* is symbolic of the Japanese countryside [4]. However, *S*. *frequens* abundance has declined by 1% over the past 20 years [5], and several reports suggest that nursery-box application of fipronil is a major cause of the decline of dragonfly nymph numbers in rice paddy fields [6–7].

Fipronil and neonicotinoids are systemic insecticides designed to protect plants against insects that cause damage to crops, and they are presently used on a very large scale in the global insecticide market [8]. Neonicotinoids and fipronil, as well as several of their toxic metabolites, are taken up by roots or leaves and translocated to all parts of the plant, which effectively makes the treated plant toxic to insets. Fipronil and imidacloprid disrupt neural transmission in the central nervous system of organisms; imidacloprid binds to the nicotinic acetylcholine receptor, whereas fipronil inhibits the GABA receptor. Both insecticides produce lethal as well as a wide range of sublethal adverse impacts on invertebrates and some vertebrates [8–9]. The effectiveness and ease of use of imidacloprid and fipronil as insecticides have contributed to their increase in use for rice cultivation in Japan, particularly for nursery-box applications.

The transition in Japanese agriculture to using systemic insecticides fipronil and imidacloprid has generated additional concern about the role of insecticides in the decline of dragonflies. Although research investigating the causes of large-scale dragonfly losses is extensive [10–11], assessment of the risks posed by sublethal exposure to insecticides is limited by the lack of efficient methods to detect and quantify those effects. Specific guidelines for the use and registration of agrichemicals in Japan are mandated by Japan’s Agricultural Chemicals Regulation Law and the Ministry of the Environment determines the level of exposure that poses a hazard to ecosystems [12]. In the current paradigm, toxicity values are established for individual compounds in a three-tier system that first tests acute contact toxicity based on test guidelines of the Organization for Economic Co-operation and Development [13]. Presently, mortality is the only measured endpoint, and data on sublethal effects are not required for insecticide registration. Despite the focus on lethal effects, sublethal insecticide concentrations may negatively influence population dynamics and activity of non-target aquatic and terrestrial animals as reviewed by others [14–17]. The need for improved methods to predict sublethal behavioral risks has gained recognition [18–19], and one important sublethal pathway is behavior, including locomotion and feeding activity [20–27].

Recent studies have identified a variety of pesticides to which aquatic organisms are exposed depending on application period due to contamination of running and irrigation water environments [28–32]. Among these, imidacloprid and fipronil were nearly ubiquitous on the paddy soil surface and in paddy water immediately after the transplantation at concentrations of 278.4 μg/kg in soil and 30.2 μg/L in water for imidacloprid [33] and 65.8 to 92.1 μg/kg in soil and 0.9 to 2.5 μg/L in water for fipronil [34]. Imidacloprid has a relatively high water solubility of 514 mg/L [35–36] and has a high potential to leach into the aquatic environment [16, 32]. The concentration of fipronil sulfone both in paddy water and soil was relatively high during the first 35 d after transplanting, and the dissipation rates in both media were lower than those of fipronil [34]. Moreover, fipronil and imidacloprid accumulate in paddy soil following multi-year applications [37]. Although acute toxicity assessments of not only fipronil but also its metabolites are needed to determine their effects on dragonflies, there is no known toxicity data at sublethal levels for *Sympetrum* spp. Most recent research has focused on bees and sublethal effects of neonicotinoids on honey bees at many different physiological levels [26, 38–39]. These studies provide a better understanding of the mechanisms of toxicity of these insecticides in invertebrates.

According to standard application methods specified by insecticide manufacturers, these insecticides are applied to nursery-boxes only once before transplantation and continue to control pests for several months via systemic or slow effects. Fipronil and imidacloprid concentrations in paddy water and soil reach a maximum and dissipate to half within 3 d after transplantation [33–34, 40–41], but these compounds tend to remain stable in the soil at high concentration [6]. Meanwhile, the eggs of *S*. *frequens* and *S*. *infuscatum* are likely to hatch within about 2 d after flooding the rice paddy just before transplantation [42]. Prolarva molt to the next instar (instar 2) in 20 min. Thus, instar 2 larvae are expected to be exposed to high concentrations of these insecticides after transplantation. Furthermore, larvae generally spend 60 d from hatching to emergence (molting 10 times with around 5 d at each instar) [43]. Therefore, *S*. *frequens* and *S*. *infuscatum* larvae are expected to be exposed to both the maximum and lowest concentrations of insecticides during the larval stage.

The objectives of this study are to determine the effects of short-term exposure to sublethal insecticide concentrations on two key predatory activity traits: quantity captured and time to prey capture. Then, we investigated mortality following exposure to sublethal pulses of imidacloprid, fipronil and fipronil sulfone. Our findings indicate that sublethal effects on feeding inhibition appear to lead to severe mortality and that metabolites of fipronil have higher toxicity than fipronil on two *Sympetrum* species based on evaluation of feeding inhibition.

## Materials and methods

### Insect materials

Sexually mature *S*. *infuscatum* and *S*. *frequens* females were collected over 2 years from a paddy field at Miyagi University in Miyagi Prefecture, Japan (38°13’N, 140°49’E). Females were captured while ovipositing and held by the wings while dipping the tip of the abdomen into a glass tube to collect eggs. In 2010, a total of 2,518 *S*. *infuscatum* eggs were collected from 20 females in a dry glass tube. In 2011, a total of 4,518 *S*. *frequens* eggs were collected from 20 females in distilled water. Eggs collected from each species were combined at the end of collection. The eggs were allocated (50 eggs per pack) into water-permeable packs containing soil that had been oven-dried at 110°C for 24 h. These packs of *S*. *infuscatum* and *S*. *frequens* eggs were placed on the surface of a paddy at the Miyagi University farm on 30 September 2010 and 12 October 2011, respectively, for overwintering in order to allow the eggs to complete diapause under natural conditions. The packs of *S*. *infuscatum* and *S*. *frequens* eggs were removed from the paddy and transported to the laboratory on 15 May 2011 and 19 May 2012, respectively. The contents of each pack were transferred to a square plastic tray (L = 10 cm, W = 10 cm, H = 3 cm), submerged in distilled water to a depth of 2 cm, and the trays were maintained in an incubator (GC351, Sanyo, Japan) at 23°C with a photoperiod of 18L:6D (light intensity = 3,000 lux). Beginning on 16 May 2011 and 20 May 2012, respectively, the eggs were examined daily under a binocular microscope (SZ60, Olympus, Japan) at 30X magnification. Newly hatched larvae were counted to determine the day of hatching in each experiment. Instar 2 larvae were reared individually in cells (1 cm diameter, 1.5 cm high) filled with distilled water. Each individual was fed with a brine shrimp (*Artemia* sp.) before use in experiments to avoid starvation. We confirmed that all larvae succeeded in feeding on the brine shrimp. Then, larvae were moved to the acute toxicity test vessels using a thin needle in order to prevent the transfer of organic matter. The brine shrimp and materials used in the rearing of dragon fly larvae were assumed to be free of pesticides, although analysis to confirm this was not conducted.

### Pesticide testing

Technical-grade chemicals were used in all experiments in this study. Imidacloprid, fipronil and fipronil sulfone standards (>99% purity) and analytical grade solvents used for chemical analyses were purchased from Sendai Wako Pure Chemical Industries (Sendai, Japan). Water was produced using a Milli-Q Water Purification System (Millipore, Billerica, MA, USA). For each insecticide, 200 mg of imidacloprid, fipronil or fipronil sulfone, was dissolved in 200 ml acetone to produce stock solutions. We then made test solutions at concentrations of 0.01, 0.1, 1.0, 10, 100 and 1000 μg/L (S1 Table). The stock solutions were stored at 4°C until use in experiments (within 5 d). The concentration of acetone in test solutions was 0.1% (v/v), and we confirmed that acetone at this concentration had no effect on larval mortality in the experiment.

### Acute toxicity test

Acute toxicity testing was conducted on *S*. *infuscatum* and *S*. *frequens* in 2011 and 2012, respectively, by standard acute toxicity test methods [13]. The 48-h acute toxicity tests using instar 2 larvae of *S*. *infuscatum* and *S*. *frequens* starting 24 h after hatching were conducted under static conditions in a cell (1 cm diameter, 1.5 cm high) using one larva per 3.7 ml of test solution held in an incubator at 21±1°C in the dark. During the 48-h exposure period, the larvae were not fed to avoid energetic carry-over effects due to changes in food intake during the exposure period to the post-exposure period in accordance with OECD acute immobilization test guidelines for testing chemicals with *Daphnia* sp. [13]. Test media was renewed after 24 h, and basic water chemistry parameters, pH and dissolved oxygen (DO), were measured before (0 h) and after the acute toxicity test. pH and DO measured in the control at the beginning and end of the test period showed changes in pH of 7.05±0.28 to 7.62±0.2 and DO of 8.32±0.2 to 7.82±0.28. In the acute test, physicochemical variables did not show significant increases or decreases over the experiment period.

Mortality of *Sympetrum* larva was defined as the rectum not functioning, as the pumping action of the rectum renews water in the rectum for respiration throughout larval stages [44]. Thus, pumping movements of the rectum were observed daily using a binocular microscope. The insecticides, imidacloprid, fipronil and fipronil sulfone, were tested on 24 individuals for each concentration (24 larvae × 6 concentrations; total of 144 larvae per insecticide for each species). Additionally, 24 larvae were tested as controls along with test groups for each insecticide and dragonfly species (24 larvae × 3 insecticides; total of 72 larvae for control groups for each species).

### Behavioral experiment

Dragonfly larvae detect and capture live prey items (other organisms). After instar 2, factors of prey availability, such as quantity and efficiency of prey capture, largely determine larval growth rate, size increase at the next molt and survival [45]. We considered two parameters as being important for dragonfly larvae survival in the field: acquiring sufficient prey by the next instar stage, and minimizing the costs associated with capturing prey (e.g. capturing prey in the shortest time possible). Consequently, based on brine shrimp consumption patterns by larval instar for each species [43], feeding inhibition (FI) was examined by two methods: (1) number of brine shrimp consumed by *S*. *infuscatum* (starting with 10 brine shrimp), and (2) time to capture brine shrimp by *S*. *frequens*. Briefly, previous results [43] showed that *S*. *infuscatum* instar 2 larvae consumed more brine shrimp per day than *S*. *frequens*, while *S frequens* consumed one brine shrimp per day to reach instar 3. Thus, we consider quantity of prey captured and time to capture as suitable test parameters for *S*. *infuscatum* and *S*. *frequens* larvae, respectively. To test FI, we used instar 2 larvae following exposure to each insecticide for 48 h. Larvae were moved to a dish filled with distilled water and then were moved individually to a cell (1 cm diameter, 1.5 cm high) filled with 3.7 ml of distilled water for experiments. The larvae were allowed to acclimate to the cell for 20 min before the start of the experiment.

#### S. infuscatum

**Fig 1:**
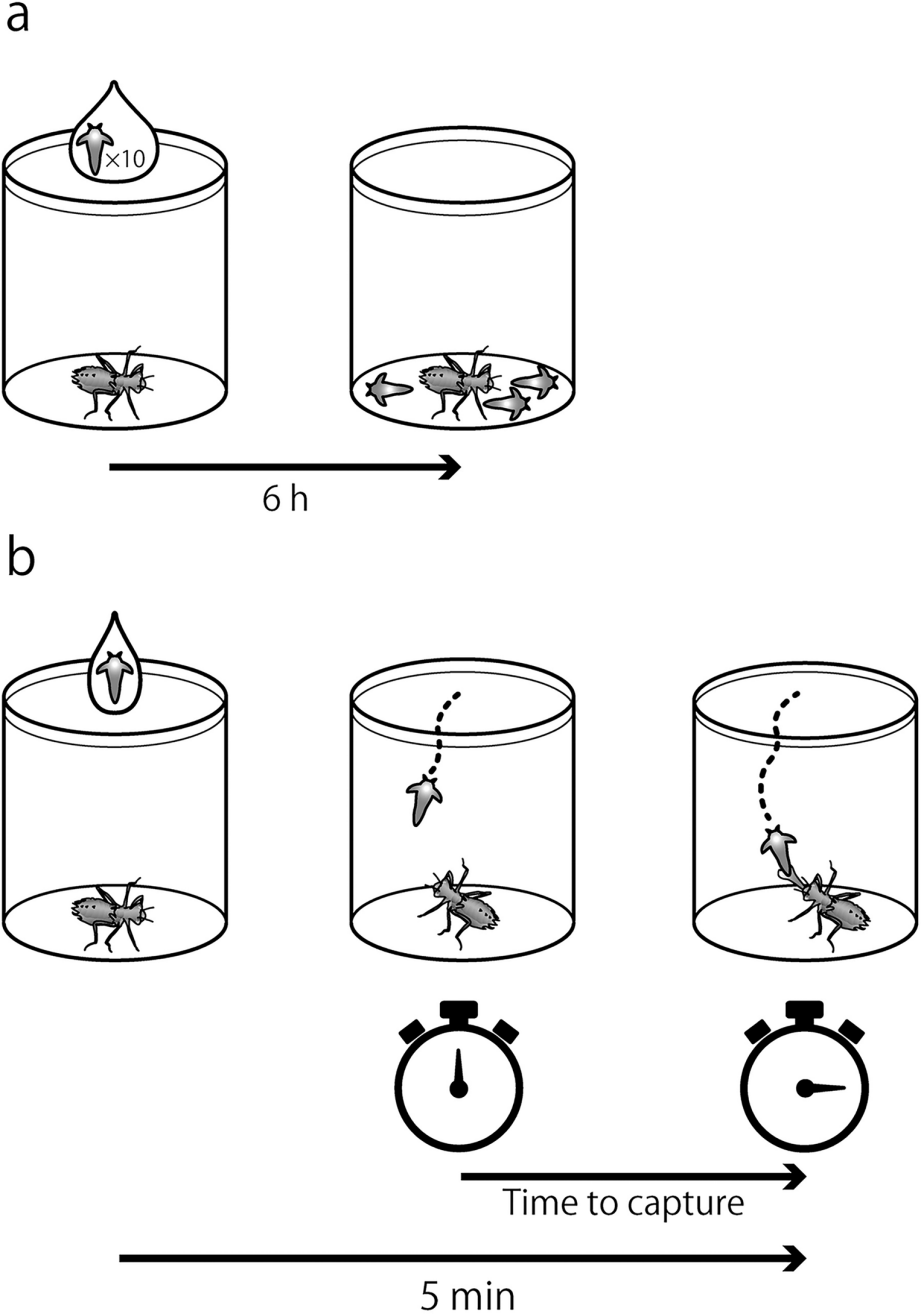
Feeding behavior experimental design. (a) Experiment to score the number of brine shrimp consumed by *S*. *infuscatum* larvae. (b) Experiment to score the time to capture a brine shrimp by *S*. *frequens* larvae.

The behavior test started with the release of 10 live brine shrimp individuals into each cell (Fig 1A). Then, micro plates were maintained in an incubator (GC351, Sanyo), at 23°C with light (light intensity = 3,000 lux). After 6 h, the number of brine shrimp remaining was scored under a binocular microscope (SZ60, Olympus, Japan). For each insecticide and the control, the test was conducted with 24 individuals, as for the acute toxicity test.

Feeding inhibition (FI) was calculated using the following equation:

$$FI=\frac{F_{con}-F_{tox}}{F_{con}}\times 100\;(\%)$$

Here, F_*con*_ is the mean number of brine shrimp consumed per larva in the control group and F_*tox*_ is the number of brine shrimp consumed per larva exposed to imidacloprid, fipronil and fipronil sulfone treatments.

#### S. frequens

Time to capture brine shrimp was measured with a single larva and a single brine shrimp gently released on the surface of the water in the cell at the start of the experiment (Fig 1B). The time to capture was taken as the time from when the larva perceived the presence of the brine shrimp recognized as bending the head or body toward the brine shrimp to when the larva preyed on the brine shrimp. The total time of observation for each larva was set to 5 min. Larval feeding activity was monitored under a binocular microscope (SZ60, Olympus). Testing was conducted with 24 individuals in each treatment and the control, as for the acute toxicity test. FI was defined as the percentage of larvae in each group that failed to capture the brine shrimp within 5 min. This test was conducted in the laboratory with an ambient air temperature of 23±0.5°C and illumination by LED light (light intensity = 2,800 lux).

### Mortality and time to reach instar 3

Following the behavior test, larvae were reared in an incubator (GC351, Sanyo) at 23°C with a photoperiod of 18L:6D (light intensity = 3,000 lux) until reaching instar 3. Larvae were reared individually in a cell (1 cm diameter, 1.5 cm high) filled with 3.7 ml of distilled water and fed about 20 brine shrimps to satiation once per day, and the remaining brine shrimp were then removed on the next day. Mortality of *S*. *infuscatum* and *S*. *frequens* larvae were monitored daily under a binocular microscope. Mortality of *Sympetrum* larva was defined as the rectum not functioning. Living larvae were also examined daily under a binocular microscope until larvae reached instar 3. The day of the molt was recorded as time to reach instar 3.

### Data and statistical analysis

For the number of brine shrimp (*Artemia* sp.) consumed by *S*. *infuscatum* and time to capture brine shrimp by *S*. *frequens*, values for individuals and means of the treatment groups were compared to the mean of the respective control and analyzed using Dunnett’s test and Steel’s test, respectively. Differences in mortality between 48-h acute toxicity test and to instar 3 were analyzed by Welch’s two sample *t*-test. The lethal concentration to 50% of the population (LC_50_) of the 48-h acute toxicity test and instar 3 larvae and effect for feeding inhibition concentration to 50% of the population (FI EC_50_) of *S*. *frequens* were estimated by probit analysis. FI EC_50_ of *S*. *infuscatum* was estimated by dose-response curve analysis. The software package R, version 3.1.1 [46] was used for all statistical analyses. Multiple comparisons were made using the “multcomp” library, version 1.1–1 [47]. Dose-response curves were made using the “drc” library, version 3.0–1 [48].

## Results

### Number of brine shrimp consumed by *S*. *infuscatum*

**Fig 2:**
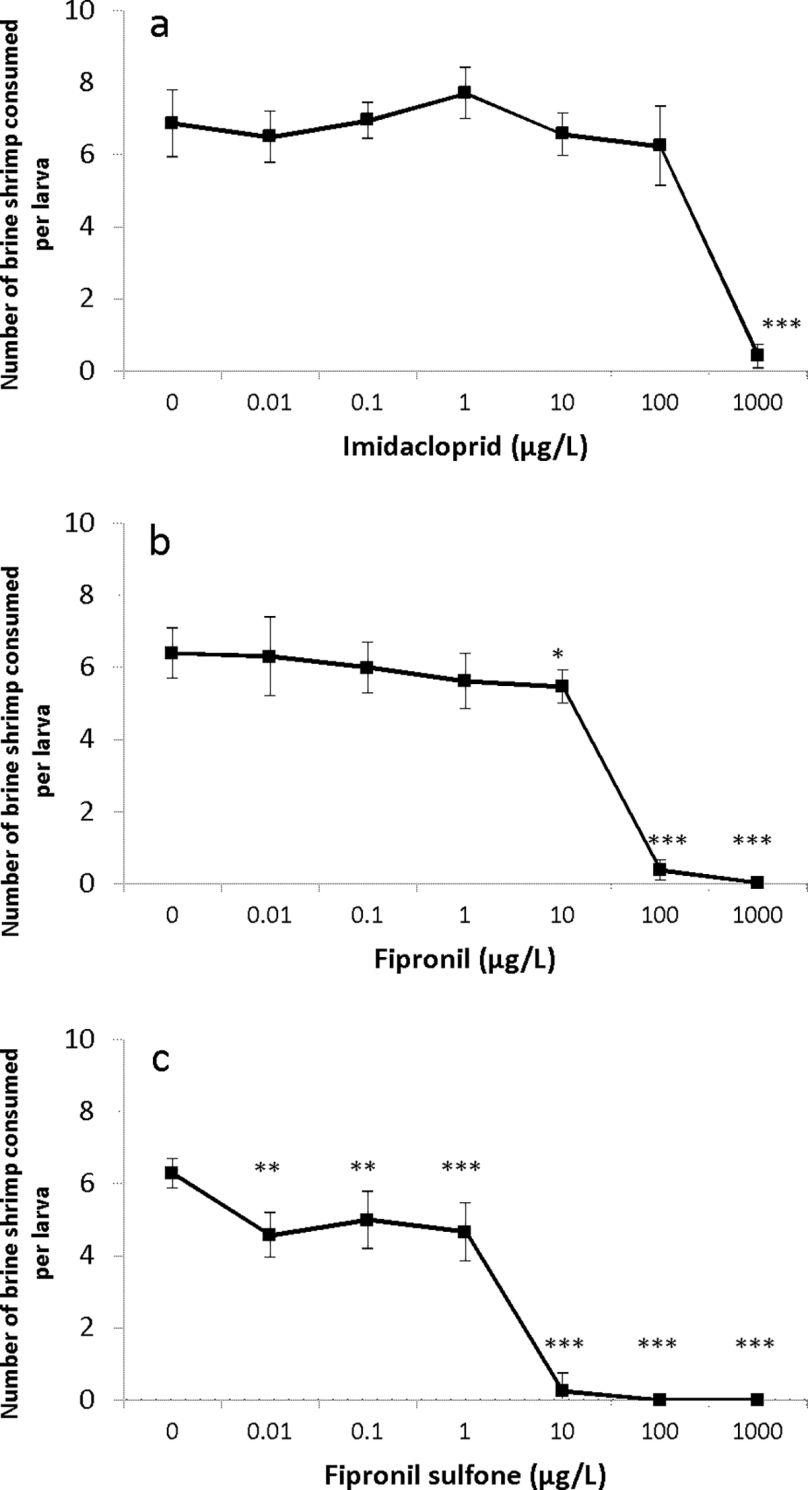
**Effect of (a) imidacloprid, (b) fipronil and (c) fipronil sulfone on feeding activity of *S*. *infuscatum* larvae after 48-h exposure.** Asterisks indicate a significant difference compared to control; *p < 0.05, **p < 0.01, ***p < 0.001.

The number of brine shrimp consumed by *S*. *infuscatum* larvae following treatment with imidacloprid at 1000 μg/L (0.4 ± 0.8) was significantly less than that of the control larvae (6.9 ± 0.9; Steel’s test, *p* < 0.001), representing a 17.3-fold decrease (Fig 2A, S2 Table). Larvae exposed to 10, 100 and 1000 μg/L fipronil also consumed significantly fewer brine shrimp (5.5 ± 0.5, 0.4 ± 0.3 and 0, respectively) than control larvae (6.4 ± 0.7) (Dunnett’s test; 10 μg/L: *p* = 0.047; 100 μg/L: *p* < 0.001; 1000 μg/L: *p* < 0.001) (Fig 2B, S2 Table). Larvae exposed to 0.01, 0.1, 1.0, 10, 100 and 1000 μg/L fipronil sulfone also consumed significantly fewer brine shrimp (4.6 ± 0.6, 5.0 ± 0.8, 4.7 ± 0.8, 0.3 ± 0.5, 0 and 0, respectively) than control larvae (6.3 ± 0.4) (Dunnett’s test; 0.01 μg/L: *p* = 0.0014; 0.1 μg/L: *p* = 0.0014; 1.0, 10, 100 and 1000 μg/L: *p* < 0.001) (Fig 2C, S2 Table).

### 48-h acute toxicity, feeding inhibition and mortality to instar 3 for *S*. *infuscatum*

#### Imidacloprid

**Fig 3:**
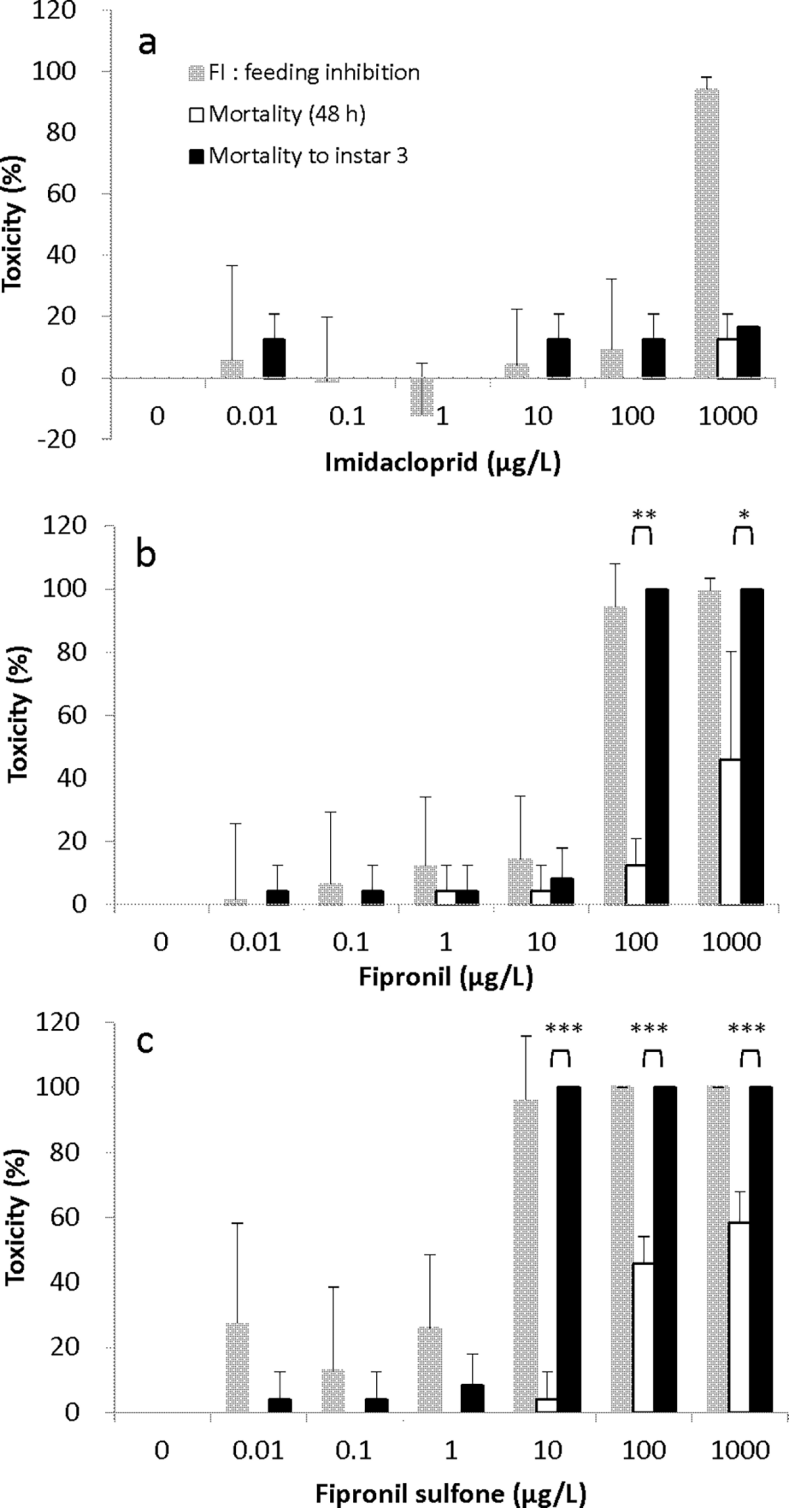
**Mortality in 48-h acute toxicity test, FI after 48-h exposure and mortality to instar 3 for *S*. *infuscatum* larvae exposed to (a) imidacloprid, (b) fipronil and (c) fipronil sulfone.** Each treatment was conducted in quadruplicate and each replicate contained six larvae. To confirm that delayed toxicity after 48-h exposure consequently causes mortality to instar 3 via feeding inhibition, we compared mortality at 48 h to that at instar 3. Asterisks indicate significant differences; *p < 0.05, **p < 0.01, ***p < 0.001.

No statistically significant differences were noted between mortality in the 48-h acute toxicity test and to instar 3 at all concentrations (Fig 3A). Mortality in the 48-h acute toxicity test in *S*. *infuscatum* larvae treated with 0.01, 0.1, 1.0, 10 and 100 μg/L was 0%, and mortality at 1000 μg/L was 12.5%. FI at 0.01, 10, 100 and 1000 μg/L was 5.5%, 4.4%, 9.2% and 94%, respectively. Larvae in the 0.1 and 1 μg/L treatments consumed more brine shrimp than larvae in the control and had FI of -1.2% and -12.2%, respectively.

#### Fipronil

The dose-dependent effect of exposure to fipronil was apparent in feeding activity. Mortality to instar 3 was significantly higher than mortality in the 48-h acute toxicity test for *S*. *infuscatum* larvae at 100 and 1000 μg/L fipronil (Welch test; 100 μg/L: *p =* 0.0012; 1000 μg/L: *p <* 0.05) (Fig 3B, S3 Table). In the highest treatment groups, 100 and 1000 μg/L, mortality in the 48-h acute toxicity test was 12.5% and 45.8%, respectively, FI was 94.1% and 99.4%, respectively, and mortality to instar 3 was 100% at the two highest treatment levels.

#### Fipronil sulfone

Mortality to instar 3 was significantly higher than mortality in the 48-h acute toxicity test at 10, 100 and 1000 μg/L fipronil sulfone (Welch test; 10, 100 and 1000 μg/L: *p <* 0.001) (Fig 3C, S3 Table). FI for larvae treated with 10, 100 and 1000 μg/L was 96%, 100% and 100%, respectively. Moreover, mortality to instar 3 was 100% at concentrations 10, 100 and 1000 μg/L.

### Time to capture brine shrimp for *S*. *frequens*

**Fig 4:**
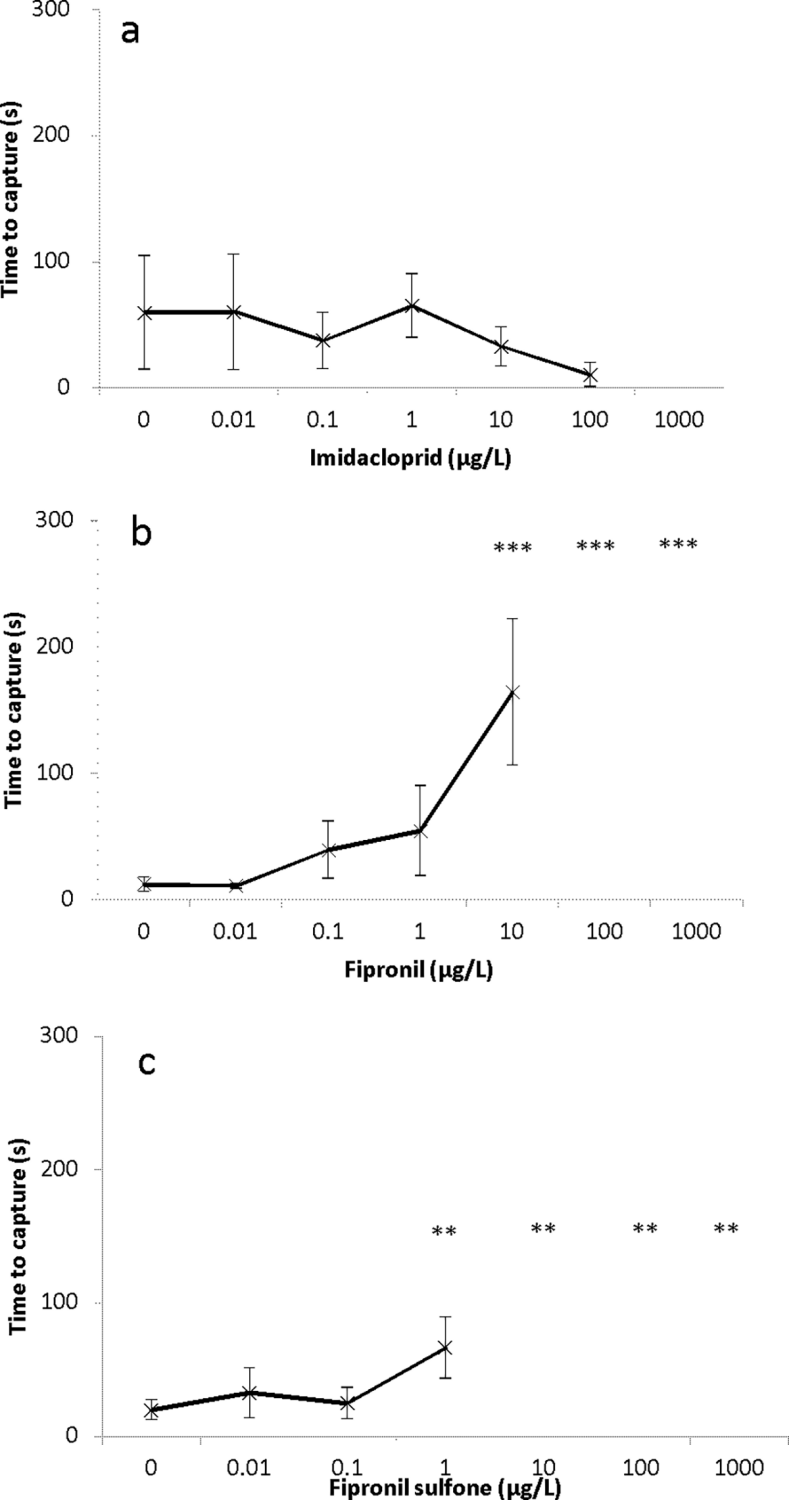
**Effect of (a) imidacloprid, (b) fipronil and (c) fipronil sulfone on feeding activity of *S*. *frequens* larvae after 48-h exposure.** Asterisks indicate a significant difference compared to control; **p < 0.01, ***p < 0.001. Time to capture in fipronil (100 and 1000 μg/L) and fipronil sulfone (10, 100 and 1000 μg/L) treatments exceeded the 5-min time limit, and Dunnett's test could not be applied to these treatments because the variance was zero due to setting the time to 5 min. Values for these treatment groups were interpreted as being significantly higher than in the control based on the 5-min time limit being much higher than the highest time to capture in the 10 μg/L (fipronil) and 1 μg/L (fipronil sulfone) treatments, which were significantly higher than the respective controls. The large variation observed particularly in the imidacloprid treatment and in the control is probably due to individual differences in locomotor activity.

No statistically significant differences in time spent to capture brine shrimp were observed between the control and groups exposed to 0.01, 0.1, 1.0, 10 and 100 μg/L imidacloprid (*F*_5_,_94_ = 0.8557, *p* = 0.514, ANOVA). However, none of the larvae in the highest group (1000 μg/L imidacloprid) consumed brine shrimp during the observation period (Fig 4A). The time to capture brine shrimp showed an increasing trend and was significantly higher than in the control for the highest concentration of both fipronil and fipronil sulfone. Steel’s test revealed that *S*. *frequens* treated with fipronil was significantly different from the control group at 10, 100 and 1000 μg/L (*p* < 0.001) (Fig 4B, S4 Table). Larvae treated with 10 μg/L fipronil spent 164.4 ± 58.0 s compared to the control, which spent 12.7 ± 5.7 s. No larvae exposed to 100 and 1000 μg/L fipronil captured a brine shrimp during the observation period. Larvae exposed to 1, 10, 100 and 1000 μg/L fipronil sulfone also spent significantly more time compared to control larvae (*p* < 0.01) (Fig 4C, S4 Table). Larvae treated with 1 μg/L fipronil sulfone spent 66.5 ± 40.0 s compared to control, which spent 20.0 ± 21.8 s. No larvae exposed to 10, 100 and 1000 μg/L of fipronil sulfone captured the brine shrimp during the observation period.

### 48-h acute toxicity, feeding inhibition and mortality to instar 3 for *S*. *frequens*

#### Imidacloprid

**Fig 5:**
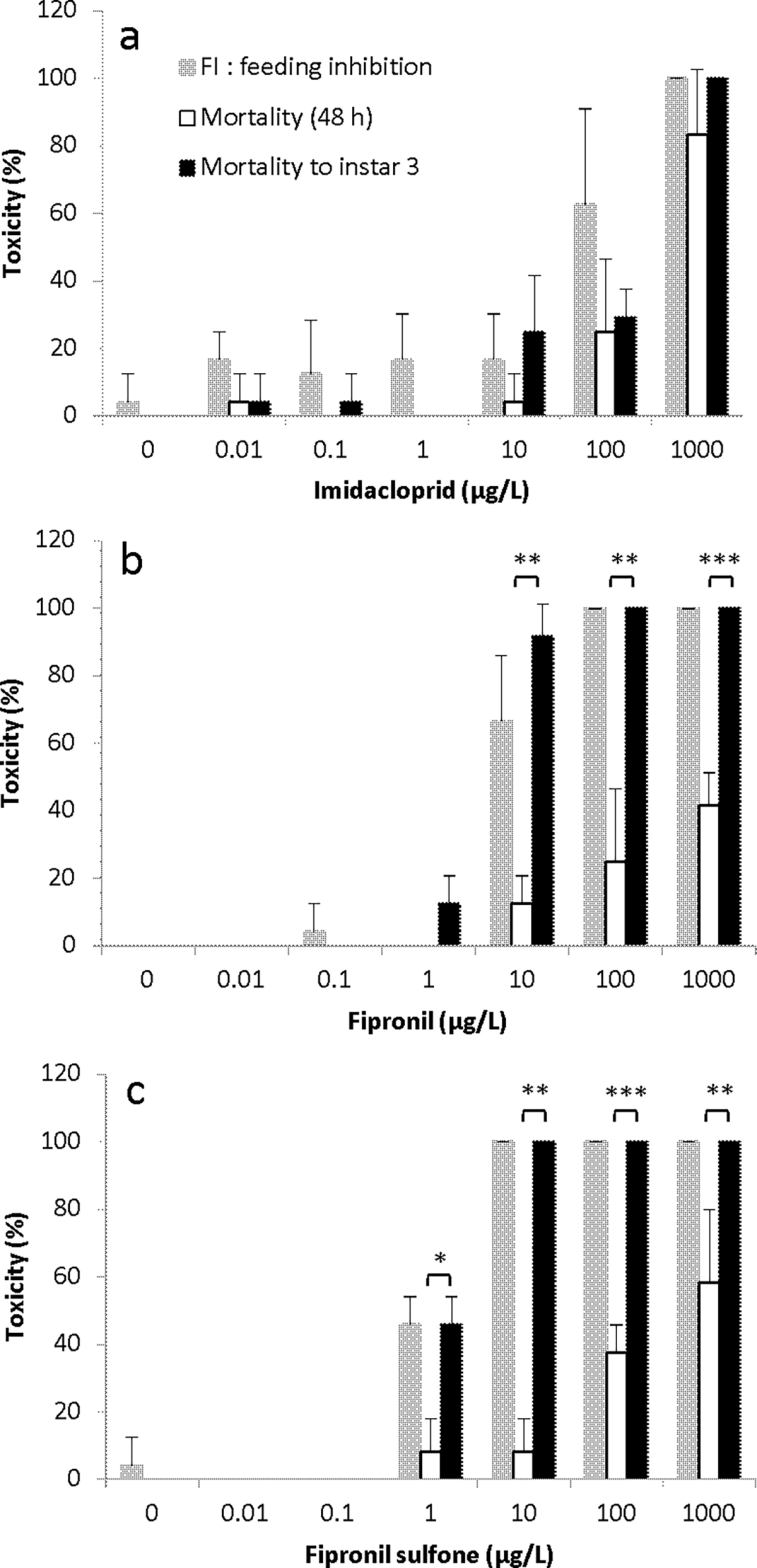
**Mortality in 48-h acute toxicity test, FI after 48-h exposure and mortality to instar 3 for *S*. *frequens* larvae exposed to (a) imidacloprid, (b) fipronil and (c) fipronil sulfone.** Each treatment was run in quadruplicate and each replicate containing six larvae. To confirm that delayed toxicity after 48-h exposure consequently causes mortality to instar 3 via feeding inhibition, we compared mortality at 48 h to that at instar 3. Asterisks indicate a significant difference; *p < 0.05, **p < 0.01, ***p < 0.001.

No statistically significant differences were noted in mortality between concentrations in the 48-h acute toxicity test and to instar 3 (Fig 5A).

#### Fipronil

Fipronil concentration had an influence on feeding activity, and mortality to instar 3 was significantly higher than mortality in the 48-h acute toxicity test for *S*. *frequens* larvae at 10, 100 and 1000 μg/L fipronil (Welch test; 10 μg/L: *p =* 0.0012; 100 μg/L: *p <* 0.01; 1000 μg/L: *p <* 0.001) (Fig 5B, S5 Table).

#### Fipronil sulfone

Mortality to instar 3 was significantly higher than mortality in the 48-h acute toxicity test at 1, 10, 100 and 1000 μg/L fipronil sulfone (Welch test; 1 μg/L: *p* = 0.015; 10 μg/L: *p* = 0.0015, 100 μg/L: *p* < 0.001; 1000 μg/L: *p* = 0.009) (Fig 5C, S5 Table). Mortality to instar 3 for *S*. *frequens* was consistent with FI (Fig 5C). The 48-h mortality of *S*. *frequens* larvae treated with 1, 10, 100 and 1000 μg/L was 4.2%, 4.2%, 37.5% and 58.3%, respectively, while FI was 41.7%, 100%, 100% and 100%, respectively, and mortality to instar 3 was 29.2%, 100%, 100% and 100%, respectively.

### LC_50_ of acute toxicity, FI EC_50_ and to instar 3 LC_50_

The 48-h LC_50_, FI EC_50_ and to instar 3 LC_50_ values are shown in Table 1. Because the dose-dependent effect of exposure to imidacloprid was not apparent in 48-h acute toxicity and feeding inhibition for *S*. *infuscatum*, LC_50_ and EC_50_ of imidacloprid were not calculated. Fipronil sulfone was highly toxic to *S*. *infuscatum*, and the LC_50_ to instar 3 was 0.8 μg/L, compared to 8.4 μg/L for fipronil. In this study, fipronil sulfone was 2.8 times (48-h LC_50_), 9.7 times (FI EC_50_) and 10.5 times (instar 3 LC_50_) more toxic to *S*. *infuscatum* than fipronil. Similarly, fipronil sulfone was 6.6 times (48-h LC_50_), 2.9 times (FI EC_50_) and 9.1 times (instar 3 LC_50_) more toxic to *S*. *frequens* than fipronil.

**Table 1:** Mortality (48-h LC_50_ and To instar 3 LC_50_) and feeding inhibition (FI EC_50_) endpoints of imidacloprid, fipronil and fipronil sulfoneto *S*. *infuscatum* and *S*. *frequens*.

		48-h LC_50_	FI EC_50_	To instar 3 LC_50_
		(μg/L)	(μg/L)	(μg/L)
		Estimate (95% CI)	Estimate (95% CI)	Estimate (95% CI)
	Imidacloprid	-	-	4130 (1370–12454)
*S*. *infuscatum*	Fipronil	1020 (421–2469)	29.3 (10.7–47.9)	8.4 (5.6–12.6)
	Fipronil sulfone	362 (164–795)	3.0 (0.7–5.2)	0.8 (0.6–1.1)
	Imidacloprid	227 (123–516)	6.7 (3.7–12.1)	41.8 (30.1–58.0)
*S*. *frequens*	Fipronil	2775 (579–13302)	2.9 (1.9–4.5)	9.1 (6.7–12.5)
	Fipronil sulfone	421 (165–1071)	1.0 (0.7–1.4)	1.0 (0.8–1.4)

LC_50_ of the 48-h acute toxicitytest and instar 3 larvae and FI EC_50_ of S. frequens were estimated by probit analysis. FI EC_50_ of S. infuscatum was estimated by dose-response curve analysis (S1 and S2 Figs).

### Time to reach instar 3

Table 2 shows the time to reach instar 3 after the 48-h exposure, as indicated by the appearance of the molt on instar 2 individuals. *S*. *infuscatum* larvae exposed to 1000 μg/L imidacloprid took significantly longer to reach instar 3 (5.9 ± 0.9) than control larvae (4.9 ± 0.3) (Steel’s test, *p* < 0.001, S6 Table). *S*. *frequens* larvae exposed to 100 and 1000 μg/L imidacloprid took significantly longer (5.8 ± 1.0 and 7.0 ± 0.0) than control larvae (5.2 ± 0.5) (Steel’s test, 100 μg/L: *p* < 0.05; 1000 μg/L: *p* < 0.001, S6 Table). *S*. *infuscatum* and *S*. *frequens* larvae exposed to 10 μg/L fipronil took significantly longer (5.5 ± 0.7 and 7.0 ± 1.4) than control larvae (4.8 ± 0.8 and 4.1 ± 0.3) (Dunnett’s test; *p* < 0.05, Steel’s test, *p* < 0.001, S6 Table). *S*. *infuscatum* larvae exposed to 1 μg/L fipronil sulfone took significantly longer (4.3 ± 0.9) than control larvae (3.7 ± 0.6) (Dunnett’s test; *p* < 0.01, S6 Table).

**Table 2:** Time to reach instar 3 after 48-h exposure.

			Time (±SD) to reach instar 3 (days)			
Treatment	Imidacloprid		Fipronil		Fipronil sulfone	
(μg/L)	*S*. *infuscatum*	*S*. *frequens*	*S*. *infuscatum*	*S*. *frequens*	*S*. *infuscatum*	*S*. *frequens*
*Control*	*4*.*9 ± 0*.*3*	*5*.*2 ± 0*.*5*	*4*.*8 ± 0*.*8*	*4*.*1 ± 0*.*3*	*3*.*7 ± 0*.*6*	*5*.*0 ± 1*.*0*
	*n = 24*	*n = 24*	*n = 24*	*n = 24*	*n = 24*	*n = 24*
0.01	4.9 ± 0.4	5.4 ± 0.5	5.4 ± 0.8	4.2 ± 0.5	4.1 ± 0.7	4.7 ± 0.9
	n = 21	n = 23	n = 23	n = 24	n = 23	n = 24
0.1	5.0 ± 0.2	5.2 ± 0.4	5.0 ± 0.8	4.4 ± 0.6	4.1 ± 0.6	4.8 ± 0.7
	n = 24	n = 23	n = 23	n = 24	n = 23	n = 24
1	5.0 ± 0.0	5.1 ± 0.3	4.5 ± 0.7	4.1 ± 0.3	**4.3 ± 0.9**	4.9 ± 0.8
	n = 24	n = 24	n = 23	n = 21	n = 22	n = 11
10	5.0 ± 0.2	5.6 ± 0.9	**5.5 ± 0.7**	**7.0 ± 1.4**	-	-
	n = 21	n = 18	n = 22	n = 2		
100	5.0 ± 0.0	**5.8 ± 1.0**	-	-	-	-
	n = 21	n = 17				
1000	**5.9 ± 0.9**	**7.0 ± 0.0**	-	-	-	-
	n = 19	n = 4				

"n" indicates the number of individuals. Values for control groups are in italics. The highest concentrations at 100 and 1000 μg/L fipronil and 10, 100 and 1000 μg/L fipronil sulfone could not be tested due to 100% mortality before reaching to instar 3. Bold values are significant difference compared to control.

## Discussion

Previous 48-h exposure to a sublethal concentration of fipronil and fipronil sulfone drastically changed behavioral foraging traits in two ways: larvae consumed fewer brine shrimp or took longer to capture them. Body size of instar 2 larvae of *S*. *infuscatum* and *S*. *frequens* were smaller in head width by 0.6 mm and 0.4 mm, respectively, and in body length by 1.7 mm and 1.6 mm, respectively [43]. In this study, *S*. *infuscatum* larvae exposed to a sublethal concentration of fipronil sulfone demonstrated insufficient feeding behavior and consequently had high mortality to instar 3 (Fig 3C). Similarly, a significant reduction in prey capture performance for *S*. *frequens* was observed at sublethal concentrations of fipronil sulfone, suggesting that lack of feeding caused the increased mortality to instar 3 (Fig 5C). We suggest, for the first time, that fipronil and fipronil sulfone at sublethal concentrations cause delayed mortality due to feeding inhibition. These latent effects are very serious compared to the acute toxicity effect as species composition and abundance of larval assemblages of dragonflies are strongly affected by predation, including inter-species interactions [49–50]. Therefore, feeding inhibition could constitute a serious obstacle to completing the life history of predatory insects, such as dragonflies.

Several studies have reported the high toxicity of fipronil metabolites on aquatic organisms, e.g., mosquito [51], crayfish [52], diptera [53] and crustaceans [28]. For example, fipronil sulfone and fipronil sulfide are 6.6 and 1.9 times, respectively, more toxic to freshwater invertebrates than fipronil [54]. In the present study, compared to fipronil, fipronil sulfone was up to 10.5 times (instar 3 LC_50_) more toxic to *S*. *infuscatum* and 9.1 times (instar 3 LC_50_) more toxic to *S*. *frequens* (Table 1). Maul et al. [53] demonstrated that the sublethal response to fipronil in *Chironomus tentans* (diptera), particularly the immobilization endpoint, is typically more sensitive than the lethal endpoints. Immobilization EC_50_ of fipronil and fipronil sulfone are 1.3 and 3.2 times more toxic than LC_50_. Similarly, sublethal responses such as feeding activity in both *Sympetrum* spp. are more sensitive than the lethal endpoints.

The toxic action of fipronil is due to its activity as a non-competitive ɤ-amino-butyric acid (GABA) gated chloride channel blocker, leading to insect death by neuronal hyperexcitation and paralysis [55]. Fipronil sulfone is also a potent inhibitor of GABA receptors, desensitizing and non-desensitizing glutamate-activated chloride channels and is much more potent than fipronil in blocking the desensitizing currents [56]. Endosulfan, an organochlorine insecticide, is also an antagonist of the GABA receptor in the inhibitory synapse of signal transduction. Blocking the chloride channels results in overstimulation of the central nervous system. Both of endosulfan and fipronil are classified in the same insecticide group because they are GABA-gated chloride channel blockers [57]. Several studies have shown a desensitized or sensitized effect of endosulfan at sublethal concentrations to dragonflies. Previous exposure (24 h) to sublethal concentrations of endosulfan caused slower swimming speed for damselfly *Enallagma cyathigerum* [22]. However, larvae instead showed higher activity in walking and reorienting. Endosulfan also directly decreased food ingestion of the damselfly *Coenagrion puella* under predation risk [20]. Thus, paralysis mechanisms may explain these maladaptive activity changes following insecticide pulses. Accordingly, paralysis due to fipronil and fipronil sulfone sublethal effects may cause feeding inhibition post-exposure with latency of effects, which leads to death in both *Sympetrum* spp. Indeed, several reports suggest that dragonflies would be eliminated in a paddy field with realistic fipronil application levels [58–59]. Microcosm experiments showed that fipronil application to rice paddy fields resulted in the complete elimination of *S*. *infuscatum* and *S*. *frequens* larvae within 9 and 14 d after insecticide application. Moreover, larvae of Libellulidae *Crocothemis servilia mariannae* and *Orthetrum albistylum speciosum* decreased in abundance after fipronil insecticide application in mesocosm experiments [7, 37]. These results indicate that the adverse effects of fipronil and its metabolites, such as acute toxicity and delayed effects (mortality to instar 3 via feeding inhibition) are present in dragonflies in the field.

A critical factor in investigations of laboratory-based observations of insecticide actions on *Sympetrum* larvae behavior or physiology is whether the concentration-dependent effects fall within the range of concentrations that are encountered in the field. Table 3 summarizes the maximum paddy water and soil concentrations of imidacloprid, fipronil and fipronil sulfone reported in the literature. Our results suggest that mortality due to feeding inhibition can be expected to occur from environmental exposure of *Sympetrum* spp. larvae to fipronil and fipronil sulfone in the cases referenced in the literature. In the present study, the FI EC_50_ to fipronil sulfone was estimated at 3.0 and 1.0 μg/L and the LC_50_ to instar 3 was estimated at 0.8 and 1.0 μg/L for *S*. *infuscatum* and *S*. *frequens*, respectively (Table 1). These concentrations are below the maximum concentrations of fipronil sulfone in paddy soil, which range from 9.7 to 59.2 μg/kg [34]. Thus, larvae in paddy fields with insecticide application at representative application levels face increased risk of feeding inhibition and consequent mortality to instar 3. For *S*. *infuscatum* and *S*. *frequens*, FI EC_50_ for fipronil was estimated to be 29.3 and 2.9 μg/L, respectively, and to instar 3 LC_50_ was estimated to be 8.4 and 9.1 μg/L, respectively (Table 1). These concentrations are also lower than the maximum concentrations of fipronil in paddy soil, which ranged from 65.8 to 92.1 μg/kg [34], and a notably higher concentration of 192 μg/kg [37] with the application at 10,000 g/ha as part of a commercial formulation. Ecological impacts, and therefore risks, of imidacloprid and fipronil on dragonfly nymph communities depend more on soil residues than they do on waterborne residues [6]. Hence, for *S*. *infuscatum* and *S*. *frequens* larvae, short exposure to realistic field concentrations of fipronil and fipronil sulfone in the soil could easily cause feeding inhibition and consequent mortality to instar 3. However, the maximum concentrations of fipronil (range 0.9 to 2.5 μg/L) [34] and fipronil sulfone (range 0.4 to 0.9 μg/L) [34] in paddy water lie below the EC_50_ and LC_50_. *Sympetrum* larvae are usually covered with mud in the paddy surface soil during the larval stage, making them more vulnerable to fipronil and fipronil sulfone in soils than in water due to high absorption properties to soil.

**Table 3:** Maximum water and soil concentrations of imidacloprid, fipronil and fipronil sulfone in rice paddy fields sampled in previous studies with mesocosm and microcosm paddy experiments. These data are for concentrations following application at 10,000 g/ha as a commercial formulation.

	Imidacloprid	Fipronil	Fipronil sulfone
Water (μg/L)	30.2 [33]		
	39 [37]		
	49 [71]	1.0 [71]	
	52.8 [58]	1.3 [58]	
	58.6–73.9 [40]		
	189 [41]		
	240 [72]		
		0.9–2.5 [34]	0.4–0.9 [34]
	157.5 [73]		
Soil (μg/kg)	278.4 [33]		
	20 [37]		
		192 [71]	
	227.2–276.2 [40]		
		65.8–92.1 [34]	9.7–59.2 [34]
	13.55 [73]		

Imidacloprid showed higher toxicity than fipronil and fipronil sulfone for *S*. *frequens* in the acute toxicity test (Table 1). The toxicity data presented here contributes to the toxicological data for dragonfly species for the widely used neonicotinoid imidacloprid. The LC_50_ estimate for *S*. *frequens* determined here suggests that this species is highly sensitive to imidacloprid as compared to other dragonflies (1.245 ppm; *Lestes unguiculatus*, 1 ppm; *Anax junius*, 0.865 ppm; *Plathemis lydia*) [25], although comparisons are difficult because none of the experiments were conducted using the active ingredients of imidacloprid in a 48-h acute toxicity test.

The importance of considering sublethal endpoints as done in the present study is corroborated by other studies noting detrimental behavioral changes in aquatic insects due to imidacloprid exposure. Other researchers have observed sublethal endpoints including diminished feeding rate [21, 60], decreased locomotion and ventilation [61] and immobilization [62–63]. We found that sublethal exposure of *S*. *frequens* to imidacloprid at all concentrations impaired natural foraging behavior compared to that in control larvae (Fig 5A). Consistent with our results, short (12 or 48 h) exposure pulses to imidacloprid caused feeding inhibition in mayflies and black tiger shrimp [64–65]. Feeding inhibition from sublethal exposure to imidacloprid similarly appeared to be responsible for decreases in the growth and body size of the shredder, *Gammarus pulex* [66]. Further, many unfavorable effects of imidacloprid and other neonicotinoids on terrestrial insects are related to behavior; for example, observe foraging and flight ability deficits observed in bees [27, 67–70].

Imidacloprid is susceptible to runoff, and maximum concentrations have been reported within the range of 13.55 to 278.4 μg/kg [33, 37, 40, 73] in paddy soil, and from 30.2 to 240 μg/L [33, 37, 40–41, 58, 71–73] in paddy water (Table 3). The 48 h LC_50_, FI EC_50_ and to instar 3 LC_50_ to imidacloprid for *S*. *frequens* were estimated to be 227, 6.7 and 41.8 μg/L, respectively. These estimated concentrations lie below the maximum concentrations of imidacloprid, ranging from 272.2 to 278.4 μg/kg [33, 40] in paddy soil. Hence, for *S*. *frequens* larvae, short-term exposure to concentrations of imidacloprid reported in field samples could cause mortality due to acute toxicity and delayed effects through feeding inhibition. The maximum concentration of imidacloprid in water and soil in the paddy field is highly variable due to its high water solubility. However, all maximum concentrations of imidacloprid in paddy water could thus be expected to cause feeding inhibition. Higher temperatures accelerate the onset of imidacloprid toxicity [63]. Thus, a paddy field used for warming water could possibly increase the vulnerability of the larvae to the effects of imidacloprid.

Neonicotinoid compounds act as agonists of nicotinic acetylcholine receptors (nAChRs) [74–75] and cause persistent activation of cholinergic receptors, leading to hyperexcitation and eventual death [76]. In this study, sublethal 48-h exposure to all treatments of imidacloprid (0.01–1000 μg/L) elicited the opposite effects on feeding activity and led to higher mortality to instar 3 than for 48-h exposure. Our results for *S*. *frequens* exposure to imidacloprid are consistent with the effects demonstrated by others on bees. Indeed, neonicotinoid insecticide effects on bees initially cause hyperexcitation, increasing precise function, increasing motor activity and elevating body temperature before leading to hypoactivity and behavioral depression [26–27, 38, 77]. In other words, imidacloprid elicited the opposite effects on motor and precise function with elapsed time after exposure. For example, thiamethoxam sublethal exposure to acute and chronic doses elicited opposite effects on foraging flight abilities: bees were excited and flew farther in longer flights shortly after acute exposure (40 min), but flew more slowly over shorter distances after longer exposures (1 and 2 d) [26]. Likewise, 1- to 2-d exposure to thiamethoxam also impaired forager motor functioning by reducing flight duration, distance, mean velocity, and maximum velocity [27]. Therefore, 48-h exposure to imidacloprid for *S*. *frequens* may correspond to chronic exposure levels as observed for bees.

Meanwhile, imidacloprid has a hyperexcitation effect on feeding activity at 0.1 and 1.0 μg/L level of exposure in *S*. *infuscatum*, but an opposite effect at the highest concentrations (Fig 3A). Similar results have been documented in other insects, such as mayflies and bees [21, 23, 78]. The hyperexcitation effect may be indicative of nicotinic activation by low concentrations, whereas hypoactivity may be associated with metabolite toxicity at higher concentrations [77, 79, 80]. Thus, low concentrations of active substance were not high enough to induce feeding inhibition or mortality, while high concentrations induced feeding inhibition and mortality due to the toxicity of both the parent and its metabolite compound in *S*. *infuscatum* tissues. Similarly, compatibility between fipronil and fipronil sulfone metabolized in larval tissue may cause the same additive effect post-exposure in fipronil treatment.

Other mechanisms may explain these maladaptive activity changes after imidacloprid insecticide pulses. A likely explanation for the overall increased activity levels after imidacloprid insecticide exposure is that the larvae undertook more active foraging in response to higher energetic needs. Systemic insecticides increase energy consumption and requirements, as well as metabolism [39, 81]. The response mechanism mediating neonicotinoid tolerance may disrupt *Sympetrum* larvae energy metabolism due to increased energy demand for detoxification as in bees [82]. However, increased feeding activity at 0.1 and 1.0 μg/L imidacloprid did not contribute to an acceleration of larval development (Table 2). Similarly, imidacloprid at 100 and 1000 μg/L sublethal concentrations, which caused significant reduction in feeding activity, delayed larval development to instar 3 for both *Sympetrum* spp. Fipronil and fipronil sulfone at 10 and 1 μg/L sublethal concentrations also delayed larval development for both *Sympetrum* spp. These delays in larval development due to feeding inhibition may result in hampered larval development and unsuccessful emergence [4, 59].

Our study adds the insight that sublethal fipronil and fipronil sulfone may increase mortality by making dragonflies more vulnerable to feeding inhibition. Ecological risk assessment evaluations that ignore these indirect effects of insecticide exposure in the post-exposure period likely underestimate the response of predatory insects to sublethal insecticide pulses. The impact of insecticide exposure at the sublethal level should not be underestimated as these effects may induce physiological impairment that ultimately results in the loss of feeding activity and overall increased mortality. Fipronil and its metabolites have been detected during the pesticide application period in a river that flows through a region with many paddy fields [28] [83]. Importantly, use of these insecticides in the paddy fields, even at sublethal concentrations, may cause adverse effects on aquatic insects in waterways distant from the point of application.

Sensitive screening methods are needed to improve risk assessment, and identification of feeding inhibition has the potential to identify problematic compounds for further testing in order to adequately evaluate the hazards of agrichemicals to predatory aquatic insects. Thus, additional research is needed to develop new exposure test methods to evaluate lethal and sublethal effects of insecticide compounds. First, acute toxicity tests including of bottom soil sediment are needed to assess the impacts of fipronil and its metabolites because fipronil is readily absorbed by soil and several aquatic insects have life history stages in muddy environments and a predation strategy involving microbial sludge. Second, bioaccumulation tests should be examined. Finally, studies need to examine the degree and variation of insecticide sensitivity in dragonfly species using the feeding inhibition test.

Reduction of feeding activity in dragonflies may have a serious impact on the ecosystem. Libelludiae larvae are important predators of insects such as mosquitoes, which are recognized as pests and disease vectors [84–85], and consequently Libelludiae larvae are often exposed to insecticide pulses targeting the harmful insects. Evaluating the effects of these insecticide pulses is therefore highly relevant when assessing the potential of Libelludiae larvae as biocontrol agents [86–87].

## Supporting information

S1 Fig Dose-response curve for *S*. *infuscatum* in the FI test of fipronil. (PDF)

S2 Fig Dose-response curve for *S*. *infuscatum* in the FI test of fipronil sulfone. (PDF)

S1 Table Comparison of nominal and actual imidacloprid, fipronil and fipronil sulfone levels. Data are presented as mean ± standard deviation (SD).
(PDF)

S2 Table *t*-value, degrees of freedom and *p*-value in Fig 2. (PDF)

S3 Table *t*-value, degrees of freedom and *p*-value in Fig 3. (PDF)

S4 Table *t*-value, degrees of freedom and *p*-value in Fig 4. (PDF)

S5 Table *t*-value, degrees of freedom and *p*-value in Fig 5. (PDF)

S6 Table *t*-value, degrees of freedom and *p*-value in Table 2. (PDF)
