# Supplementary figures and images for: Effects of short-term, sublethal fipronil and its metabolite on dragonfly feeding activity
Source: PLoS One. 2018 Jul 11;13(7):e0200299. doi: 10.1371/journal.pone.0200299 (PMC6040742; doi:10.1371/journal.pone.0200299)

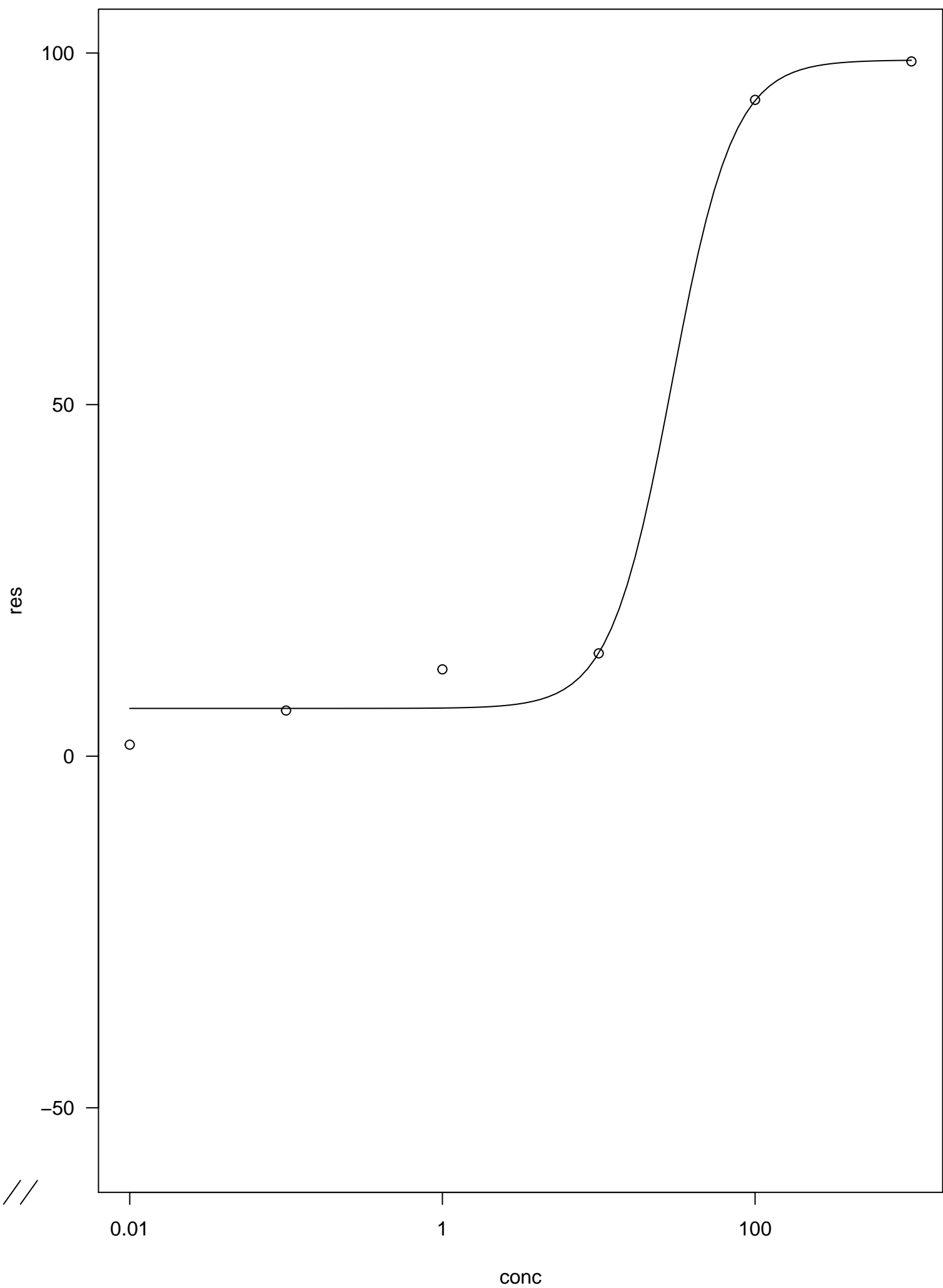

Supplement: S1 Fig — (PDF) [file pone.0200299.s001.pdf]

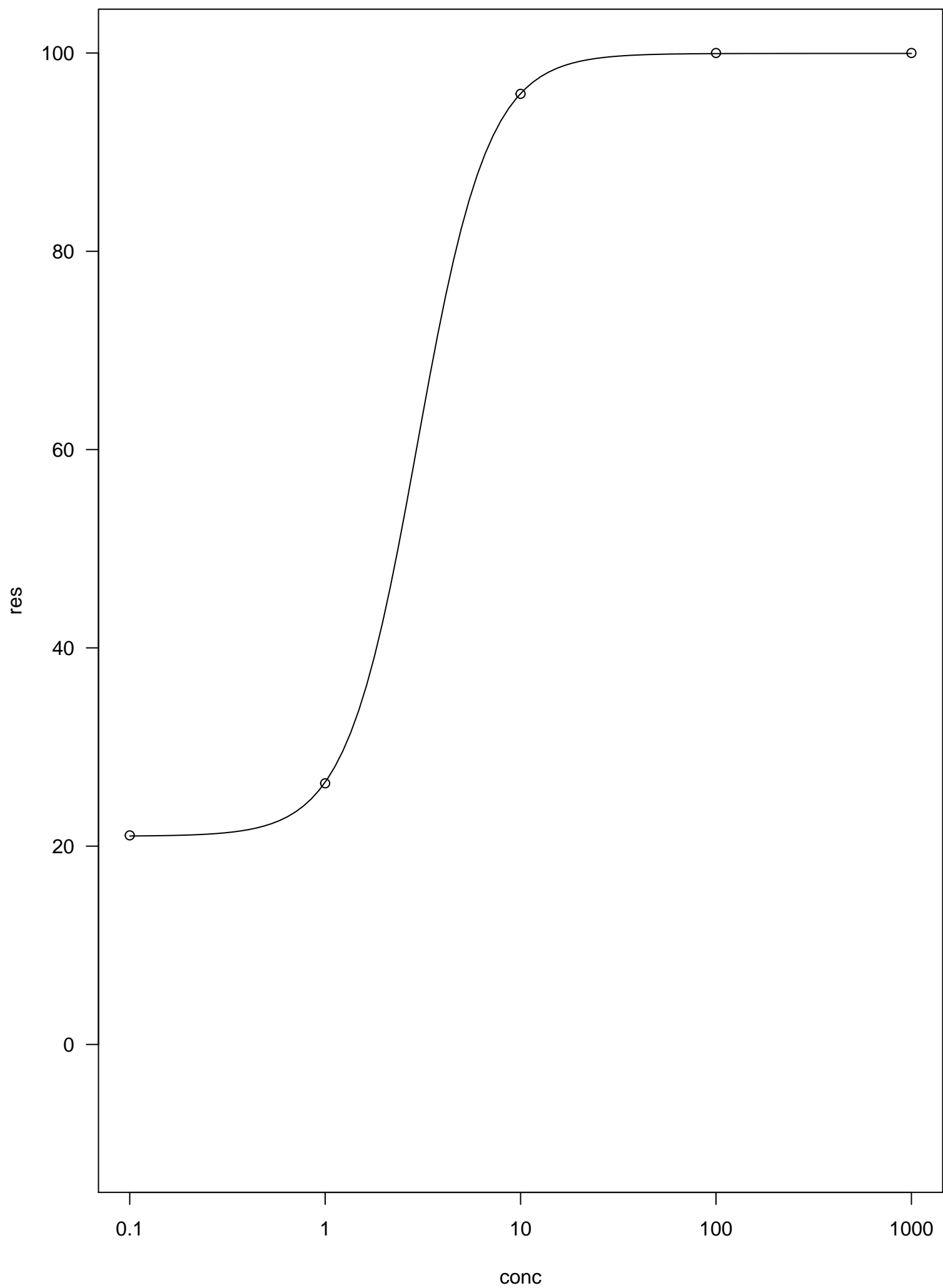

Supplement: S2 Fig — (PDF) [file pone.0200299.s002.pdf]
