# Supplementary material for: Effects of short-term, sublethal fipronil and its metabolite on dragonfly feeding activity
Source: PLoS One. 2018 Jul 11;13(7):e0200299. doi: 10.1371/journal.pone.0200299 (PMC6040742; doi:10.1371/journal.pone.0200299)
Supplement: S1 Table — Data are presented as mean ± standard deviation (SD). (PDF) [file pone.0200299.s003.pdf]

S1 Table. Comparison of nominal and actual imidacloprid, fipronil and fipronil sulfone levels at the start of traials. Data are presented as mean ±standard deviation (SD).

Imidacoprid			Fipronil			Fipronil-sulfone		
Norminal (μg/L)	Actual (μg/L)	SD	Norminal (μg/L)	Actual (μg/L)	SD	Norminal (μg/L)	Actual (μg/L)	SD
0.01	0.02	0	0.01	NA		0.01	NA	
0.1	0.1	0.01	0.1	0.1	0.01	0.1	0.1	0.01
1	1.02	0.01	1	1.03	0.03	1	1.03	0.01
10	9.96	0.08	10	9.85	0.07	10	9.75	0.07
100	98.8	0.40	100	98.5	0.70	100	97.8	0.35
1000	992.8	3.10	1000	992.5	3.50	1000	994.3	6.00

NA = not applicable.
