# Supplementary material for: Effects of short-term, sublethal fipronil and its metabolite on dragonfly feeding activity
Source: PLoS One. 2018 Jul 11;13(7):e0200299. doi: 10.1371/journal.pone.0200299 (PMC6040742; doi:10.1371/journal.pone.0200299)
Supplement: S2 Table — (PDF) [file pone.0200299.s004.pdf]

S2 Table. *t*-value, degrees of freedom and *p*-value in Fig 2.

Treatment	Imidacloprid			Fipronil			Fipronil-sulfone		
(µg/L)	*t*-value	*df*	*p*-value	*t*-value	*df*	*p*-value	*t*-value	*df*	*p*-value
0.01		n.s.			n.s.		3.743	6	< 0.001
0.1		n.s.			n.s.		3.743	6	0.0014
1		n.s.			n.s.		4.679	6	< 0.001
10		n.s.		2.615	6	0.047	17.077	6	< 0.001
100		n.s.		17.236	6	< 0.001	17.779	6	< 0.001
1000	6.026	6	< 0.001	18.817	6	< 0.001	17.779	6	< 0.001
