# Supplementary material for: Effects of short-term, sublethal fipronil and its metabolite on dragonfly feeding activity
Source: PLoS One. 2018 Jul 11;13(7):e0200299. doi: 10.1371/journal.pone.0200299 (PMC6040742; doi:10.1371/journal.pone.0200299)
Supplement: S3 Table — (PDF) [file pone.0200299.s005.pdf]

S3 Table. *t*-value, degrees of freedom and *p*-value in Fig 3.

Treatment	Fipronil			Fipronil-sulfone		
(μg/L)	*t*-value	*df*	*p*-value	*t*-value	*df*	*p*-value
0.01		n.s.			n.s.	
0.1		n.s.			n.s.	
1		n.s.			n.s.	
10		n.s.		13.952	3	< 0.001
100	11.952	3	0.0012	18.333	3	< 0.001
1000	4.443	3	< 0.05	14.162	3	< 0.001
