# Supplementary material for: Effects of short-term, sublethal fipronil and its metabolite on dragonfly feeding activity
Source: PLoS One. 2018 Jul 11;13(7):e0200299. doi: 10.1371/journal.pone.0200299 (PMC6040742; doi:10.1371/journal.pone.0200299)
Supplement: S5 Table — (PDF) [file pone.0200299.s007.pdf]

S5 Table. ***t*** **-value, degrees of freedom and** ***p*** **-value in Fig 5.**

Treatment	Fipronil			Fipronil-sulfone		
(μg/L)	*t* -value	*df*	*p* -value	*t* -value	*df*	*p* -value
0.01		n.s.			n.s.	
0.1		n.s.			n.s.	
1		n.s.		4.1369	3	0.015
10	11.952	3	0.0012	11.217	3	0.0015
100	6.58	3	< 0.01	20.333	3	< 0.001
1000	16.743	3	< 0.001	5.992	3	0.009
