# Supplementary material for: Effects of short-term, sublethal fipronil and its metabolite on dragonfly feeding activity
Source: PLoS One. 2018 Jul 11;13(7):e0200299. doi: 10.1371/journal.pone.0200299 (PMC6040742; doi:10.1371/journal.pone.0200299)
Supplement: S6 Table — (PDF) [file pone.0200299.s008.pdf]

S6 Table. *t* -value, degrees of freedom and *p* -value in table 2.

	Treatment	Imidalopride			Fipronil			Fipronil-sulfone		
	(μg/L)	*t* -value	*df*	*p* -value	*t* -value	*df*	*p* -value	*t* -value	*df*	*p* -value
S. infuscatum	0.01		n.s.			n.s.			n.s.	
	0.1		n.s.			n.s.			n.s.	
	1		n.s.			n.s.		3.013	4	< 0.01
	10		n.s.		3.013	4	< 0.05			
	100		n.s.							
	1000	4.641	3	< 0.001						
S. frequens	0.01		n.s.			n.s.			n.s.	
	0.1		n.s.			n.s.			n.s.	
	1		n.s.			n.s.			n.s.	
	10		n.s.		3.678	6	< 0.001			
	100	2.579	6	< 0.05						
	1000	3.696	6	< 0.001						
